# Package leaflets of the most consumed medicines in Portugal: safety and regulatory compliance issues. A descriptive study: Bulas dos medicamentos mais consumidos em Portugal: aspectos de segurança e de adequação regulamentar. Um estudo descritivo

**DOI:** 10.1590/1516-3180.2013.7860023

**Published:** 2014-10-17

**Authors:** Carla Pires, Marina Vigário, Afonso Cavaco

**Affiliations:** I MSc. Doctoral student, Department of Social Pharmacy, School of Pharmacy, Research Institute for Medicines and Pharmaceutical Sciences, University of Lisbon, Lisbon, Portugal.; II PhD. Associate Professor, Center of Linguistics of the University of Lisbon, Lisbon, Portugal.; III PhD. Associate Professor, Department of Social Pharmacy, School of Pharmacy, Research Institute for Medicines and Pharmaceutical Sciences, University of Lisbon, Lisbon, Portugal.

**Keywords:** Product labeling, Consumer health information, Legislation as topic, Health communication, Drug industry

## Abstract

**CONTEXT AND OBJECTIVES:**

Package leaflets are necessary for safe use of medicines. The aims of the present study were: 1) to assess the compliance between the content of the package leaflets and the specifications of the pharmaceutical regulations; and 2) to identify potential safety issues for patients.

**DESIGN AND SETTING:**

Qualitative descriptive study, involving all the package leaflets of branded medicines from the three most consumed therapeutic groups in Portugal, analyzed in the Department of Pharmacoepidemiology, School of Pharmacy, University of Lisbon.

**METHODS:**

A checklist validated through an expert consensus process was used to gather the data. The content of each package leaflet in the sample was classified as compliant or non-compliant with compulsory regulatory issues (i.e. stated dosage and descriptions of adverse reactions) and optional regulatory issues (i.e. adverse reaction frequency, symptoms and procedures in cases of overdose).

**RESULTS:**

A total of 651 package leaflets were identified. Overall, the package leaflets were found to be compliant with the compulsory regulatory issues. However, the optional regulatory issues were only addressed in around half of the sample of package leaflets, which made it possible to identify some situations of potentially compromised drug safety.

**CONCLUSION:**

Ideally, the methodologies for package leaflet approval should be reviewed and optimized as a way of ensuring the inclusion of the minimum essential information for safe use of medicines.

## RESUMO

**CONTEXTO E OBJETIVO:**

As bulas dos medicamentos são necessárias para a sua utilização segura. Os objetivos do presente estudo foram: 1) avaliar a adequação entre o conteúdo das bulas e as especificações da regulação farmacêutica e 2) identificar os aspectos que potencialmente possam comprometer a utilização segura dos medicamentos pelos doentes.

**TIPO DE ESTUDO E LOCAL:**

Estudo descritivo qualitativo com a inclusão de todas as bulas dos medicamentos de marca dos três grupos terapêuticos mais consumidos em Portugal, analisados no Departamento de Farmacoepidemiologia da Faculdade de Farmácia da Universidade de Lisboa.

**MÉTODOS:**

Utilização de uma *checklist* para recolher os dados. A *checklist* foi validada por um processo de consenso entre peritos. O conteúdo de cada uma das bulas da amostra foi classificado em relação à adequação aos aspectos regulatórios obrigatórios, como a descrição das reações adversas, dose e frequência de administração, e à adequação dos aspectos regulatórios facultativos, como a frequência das reações adversas e sintomas e procedimentos em caso de sobredosagem.

**RESULTADOS:**

Foram identificadas 651 bulas. Em termos gerais, todas as bulas foram consideradas conformes em relação aos aspectos regulatórios obrigatórios. No entanto, os aspectos regulatórios opcionais foram descritos em apenas cerca de metade da amostra de bulas, o que permite a identificação de situações susceptíveis de comprometer a utilização segura dos medicamentos.

**CONCLUSÃO:**

Idealmente as metodologias de aprovação das bulas devem ser revistas e otimizadas de forma a assegurar um mínimo de informação essencial para a utilização segura dos medicamentos.

## INTRODUCTION

Package leaflets are fundamental for the safe and effective use of medicines.^1^^,^^2^ Amongst other issues, the European Medicines Agency is responsible for the publication of legal requisites regarding the content of the package leaflets of medicinal products for human use, such as their template, the Quality Review of Documents for Human Product Information.^2^

According to the Quality Review of Documents for Human Product Information, all package leaflets must include an initial content list and be structured in six sections (Table 1).^2^ All versions (1 to 9) of the Quality Review of Documents for Human Product Information template,^2^ except the first two, have required the presence of the abovementioned content list.^3^ The first version of the Quality Review of Documents for Human Product Information was published by the European Medicines Agency in 1996; however, the United States Food and Drug Administration had already been recommending the use of package inserts for oral contraceptives, intrauterine contraceptives devices and estrogens since 1977.^2^^,^^3^^,^^4^ Package leaflets met resistance and criticism in the past, because at that time their use was associated with problems in the physician-patient relationship and in the production and distribution of medicinal products.^4^

**Table 1.**
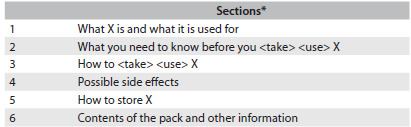
Sections of the package leaflets

^*^According to the template of the European Medicine Agency Quality Review of Documents for Human Product Information version 8^2^; X: name of medicinal products.

Usability testing was pioneered in Australia in the early 1990s, with the objective of proving the readability and comprehensibility of package leaflets.^5^ In order to ensure proper and safe use of medicines, the European Medicines Agency also developed specific regulations on usability testing for package leaflets. The first guideline of the European Medicines Agency on the readability of labels and package leaflets of medicinal products was approved in 1998 and reviewed in 2009.^6^ According to this guideline, “user testing” should consist of enrolling a group of patients with the objective of identifying comprehension issues in the content of the package leaflets; in the event that any readability problem is identified in this test, those holding marketing authorization must successively act upon the information included in the package leaflets until it is completely understood.^6^^,^^7^^,^^8^ Article 59 (3) of Directive 2004/27/EC (which amended Directive 2001/83/EC) states that the content of the package leaflets shall reflect the results of consultations with target patient groups, to ensure their readability.^9^^,^^10^ Infarmed (the Portuguese Medicines Agency) issued Law 176/2006,^11^ which takes into account the adoption of both Directives. In accordance with this law, the package leaflets should contain the following essential elements:

1. Name of the medicine (including strength and pharmaceutical form).
2. Pharmacotherapeutic category.
3. Therapeutic indications.
4. Directions or instructions for use (dosage, administration route, frequency).
5. Information on the safety of the medicine (contraindications, precautions and interactions).
6. Adverse reactions.
7. Expiration date of the medicine.
8. Qualitative composition (active substances and excipients).
9. Other relevant information, such as the duration of treatment (or temporal limits) and procedures to be adopted in cases of overdose or poisoning (e.g. emergency procedures and antidotes).

In accordance with this regulation, “other relevant information” (point 9) was classified as optional, so that the description in the content of the package leaflets would be dependent on the pharmacological characteristics of the medicinal products (e.g. package leaflets for medicines with narrow therapeutic margins must contain a description of overdose symptoms and specific procedures in cases of overdose).^9^^,^^10^^,^^11^

A brief bibliographic review, using current databases (PubMed, Academic Search Complete and Web of Science) regarding the readability of package leaflets, showed that there are problems specifically relating to the description of: 1) duration of treatment,^1^^,^^12^^,^^13^^,^^15^ 2) adverse reactions^12^^,^^16^^,^^17^^,^^18^ and 3) overdose.^19^^,^^20^ The importance of investigating the readability of package leaflets has also been confirmed in other previous studies.^3^^,^^8^^,^^20^ In addition, the use of verbal descriptors (e.g. common, rare, not known, etc.) instead of numerical descriptors (e.g. percentages, fractions, etc.) was preferred by patients for expressing the frequency of adverse reactions, with results that were more favorable than when absolute frequencies were used as an alternative to frequency ranges.^16^^,^^17^^,^^18^ Comprehension issues regarding dosage and adverse reactions were also previously identified.^12^^,^^13^ In another study, it was found that less than 20% of the participants were able to identify the symptoms of a rare life-threatening adverse reaction in a package leaflet for an antidepressant.^1^ Other issues, such as the length of the package leaflet and labeling characteristics (e.g. overdose risk due to inadequate labeling) were also considered relevant to the investigation on readability.^3^^,^^8^^,^^19^^,^^20^

Taking into consideration that the readability of package leaflets remains a controversial issue,^8^^,^^21^ that there is a lack of published studies about regulatory inconsistencies in Portuguese package leaflets and that these issues have only been evaluated by marketing authorization holders and the National Medicines Agency (i.e. not by other external institutions such as universities), the relevance of the present study became clear.

## OBJECTIVES

The aims of this study were: 1) to assess the compliance between the content of the package leaflets and pharmaceutical regulations, comprising compulsory issues, such as descriptions of dosages and adverse reactions and optional issues, such as duration of the treatment, frequency of administration and overdose symptoms; 2) to identify potential safety issues for patients; and 3) to evaluate possible qualitative inconsistencies within the sample of package leaflets and with package leaflets from other countries.

## METHODS

The package leaflets in the sample were from medicines in the three most consumed therapeutic groups within the Portuguese National Health Service (according to 2009 data),^22^ i.e. those relating to the central nervous system (Group 2 of the Portuguese prescribing guide; 33,161,500 units of medicine sold); the cardiovascular system (Group 3 of the Portuguese prescribing guide; 36,337,347 units of medicines sold); and the musculoskeletal system (Group 9 of the Portuguese prescribing guide; 14,240,989 units of medicines sold). The package leaflets of the sample were identified online in Infomed (the public database for medicines in Portugal), using the Portuguese prescribing guide’s classifications, between January and March 2012.^23^^,^^24^

The package leaflets not included were the following:

1. Package leaflets of medicinal products that are not marketed or not authorized, i.e. not available to patients.
2. Package leaflets of medicinal products used exclusively in hospitals.
3. Package leaflets of generic medicines (i.e. package leaflets very similar or equal to the package leaflets of branded products); thus, only package leaflets of branded medicines were selected.
4. Package leaflets without an initial content list (Table 1),^2^^,^^3^ which were classified as outliers and excluded from further analysis.

Firstly, each of the package leaflets in the sample was classified in relation to the type of pharmaceutical strength(s) (i.e. dose), pharmaceutical form(s) (e.g. tablets, syrups, etc.), prescription status (i.e. prescription only or over-the-counter), approval (European Medicines Agency or National Agency) and international non-proprietary name(s). Repeated package leaflets (package leaflets including the same text) were also identified through application of a manual procedure.

Secondly, the content of each of the package leaflets in the sample was classified as compliant or non-compliant with the compulsory regulatory issues (i.e. stated dosage and descriptions of all adverse reactions), and as compliant or non-compliant with the optional regulatory issues (i.e. stated frequency of adverse reactions, overdose symptoms and procedures in cases of overdose). To this end, the descriptions of these issues in the content of the package leaflets were confirmed through application of a verification list or checklist (Table 2), thus making it possible to gather data for further analysis. The abovementioned compulsory and optional issues were specifically selected because, according to the literature, these issues are have the most influence on patients’ comprehension.^12^^,^^13^^,^^14^^,^^15^^,^^16^^,^^17^^,^^18^ Three experts (two regulatory affairs experts and one epidemiologist) validated the checklist through a consensus technique (individual interviews plus mini-Delphi), taking into consideration the relevance of each of the variables in the checklist (Table 2).

**Table 2.**
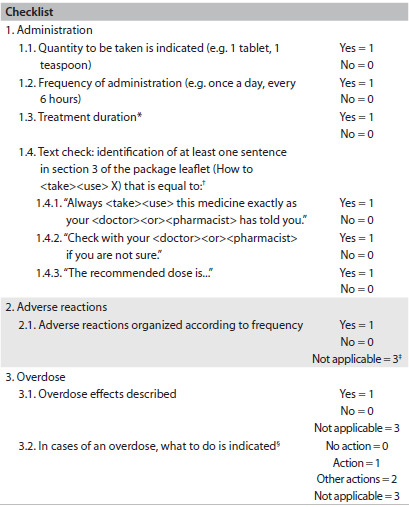
Checklist used to gather the characteristics of the package leaflets

^*^The duration of the treatment must be indicated for at least one of the medicine indications (or alternatively the minimum or maximum recommended duration); ^†^Phrases in accordance with the European Medicine Agency Quality Review of Documents for Human Product Information version 8^2^; ^‡^not applicable (package leaflets not describing adverse reactions); ^§^actions comprise: consultation with doctor or pharmacist or going to a hospital (Action = 1); other actions beyond those described in section (1) (e.g. induce vomiting, take activated charcoal, etc.) (Other actions = 2); and not applicable for package leaflets of medicines without risk of overdose (Not applicable = 3).

The results from applying the checklist were organized in an Excel file. All the data were subjected to a quality control procedure in accordance with the requisites of the NBR 5425 standard.^25^ Subsequently, package leaflets that were non-compliant (in relation to the compulsory or optional regulatory issues) were ecologically compared with equivalent Portuguese package leaflets (or package leaflets from medicines with the same active substance, pharmaceutical form and dose) and with at least one equivalent package leaflet from other countries (package leaflets selected from public databases of medicines relating to the United Kingdom, United States of America and Australia). Furthermore, the similarity between the sentences in section 3 (“How to <take><use> X”) of each package leaflet in the sample and the sentences described in section 3 of the Quality Review of Documents for Human Product Information version 8 (European Medicines Agency template) was also assessed.^2^

The Statistical Package for the Social Sciences (SPSS) for Windows (version 19.0, IBM-SPSS, Chicago, IL, USA) was used to perform the statistical analysis (descriptive statistics and chi-square test). The chi-square test was used to identify statistically significant associations (at a significant level of P < 0.05) between package leaflets from the different therapeutic groups and/or countries, taking into consideration their compliance with regulatory issues.

## RESULTS

As a consequence of applying the exclusion criteria (Table 3), 651 package leaflets were selected from the 1,072 package leaflets identified in Infomed.^23^ Thus, 421 package leaflets were excluded within the three therapeutic groups: 129 repeated; 181 not available in the database; 77 classified as outliers or not compliant with the Quality Review of Documents for Human Product Information template model;^2^ and 34 used as hospital medicines.

With regard to the composition of the medicines, the numbers of active substances mentioned in each package leaflet (and quantified in terms of a number of international non-proprietary names or generic names) were, respectively: 189 for Group 2 (or central nervous system); 128 for Group 3 (or cardiovascular system); and 81 for Group 9 (or musculoskeletal system). A total of 209 (32.1%) of the 651 package leaflets were classified as *mixed package leaflets*, i.e. package leaflets including descriptions of more than one pharmaceutical strength or pharmaceutical form. The medicines in the central nervous system group had the greatest number of different pharmaceutical forms (41), while the package leaflets in the musculoskeletal and cardiovascular system groups presented, respectively, 32 and 17. Among the 651 package leaflets, 106 (16.3%) were from over-the-counter drugs (51 in Group 2, 5 in Group 3 and 50 in Group 9).

**Table 3.**
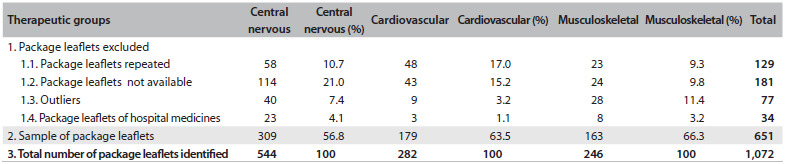
Sample of package leaflets: classification and distribution

The results from applying the checklist can be seen in Table 4. Only 218 package leaflets (33.5% of 651) described the duration of the treatment, of which 123 were in Group 2, 35 in Group 3 and 60 in Group 9. Identification of different treatment durations (including non-description) was possible within package leaflets of equivalent medicines (equal international non-proprietary names, pharmaceutical forms and pharmaceutical strength) in at least seven cases. On average, from all three therapeutic groups, the following information was found: 1) 55.6% of the package leaflets (standard deviation, SD, ± 1.9) provided the frequency of adverse reactions; 2) 61.9% (SD: ± 17.4) provided the signs and symptoms of an overdose situation; and 3) 58.8% (SD: ± 10.1) provided the recommendation that a physician or pharmacist should be consulted in cases of overdose. Also on average, only 38.1% of the package leaflets (SD: ± 3.9) provided proactive procedures for avoiding intoxication (107 in Group 2, 67 in Group 3, and 69 in Group 9), such as inducing vomiting, taking activated charcoal, lying down, contacting the poison control center and/or other actions.

**Table 4.**
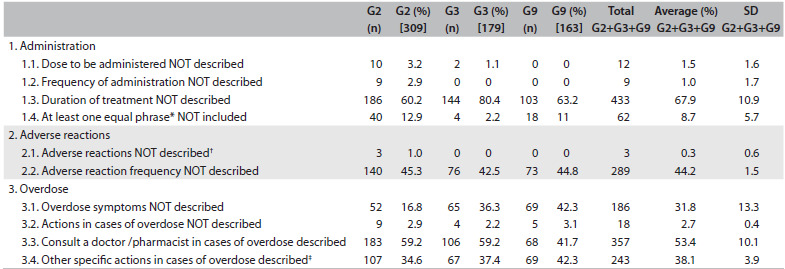
Results from checklist application

n = number of package leaflets; G = group (taking into consideration the number of package leaflets in each therapeutic group); G2 = Group 2 of the Portuguese prescribing guide, relating to the central nervous system; G3 = Group 3, relating to the cardiovascular system; G9 = Group 9, musculoskeletal system; SD = standard deviation. ^*^Package leaflets presenting sentences in accordance with what is described in points 1.4.1; 1.4.2 and 1.4.3 of the checklist (Table 2); ^†^package leaflets of medicines free from adverse reactions; ^‡^for example, induce vomiting or drink water. Note: the sum of frequencies from points 3.2, 3.3 and 3.4 do not fully correspond to 100%, since for some medicines the occurrence of overdose was not considered applicable, such as in the case of some topical forms.

In the comparison between the package leaflets of the medicines from the three therapeutic groups, the following was found: 1) the description of treatment duration (item 1.3 of the checklist) was positively associated (chi-square = 21.951; P = 0.019) with Group 3 (cardiovascular system), in comparison with the other two groups (central nervous and musculoskeletal systems); 2) absence of a description of overdose symptoms (item 3.1 of the checklist) was negatively associated (chi-square = 79.335; P < 0.001) with Group 2 (central nervous system), in comparison with the cardiovascular and musculoskeletal systems groups; and 3) absence of actions to be taken in cases of overdose (item 3.2 of the checklist) was also negatively associated (chi-square = 35.982; P < 0.001) with Group 3 (cardiovascular system), in comparison with Groups 2 and 9.

More importantly, dosage (dose and/or schedule) was omitted from 14 package leaflets (Table 5): 12 (1.8% of 651) did not describe the dose and nine (1.4% of 651) did not describe the frequency of administration. Furthermore, five of the 14 package leaflets were from medicines that need to be administered by healthcare professionals, nine were from medicines that can be self-administered by patients.

**Table 5.**
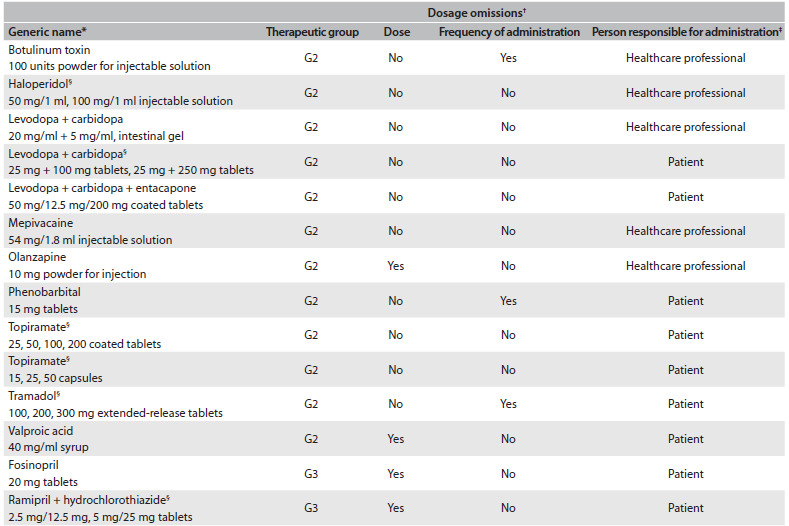
Package leaflets presenting dosage omissions

G2 = Group 2 of the Portuguese prescribing guide, relating to the central nervous system and G3 = Group 3, relating to the cardiovascular system. ^*^Each generic name corresponds to one package leaflet; ^†^Dose or frequency of administration; ^‡^Healthcare professional or patient self-administration; ^§^Mixed package leaflet.

These 14 package leaflets comprised 12 international non-proprietary names, as follows: 1) botulinum toxin; 2) haloperidol; 3) levodopa + carbidopa; 4) levodopa + carbidopa; 5) levodopa + carbidopa + entacapone, 6) mepivacaine; 7) olanzapine; 8) phenobarbital; 9) topiramate; 10) topiramate; 11) tramadol; 12) valproic acid; 13) fosinopril; and 14) ramipril + hydrochlorothiazide. Out of the 14 package leaflets, 12 were in the central nervous group and two in the cardiovascular group.

Relative to each of the 14 package leaflets without dose description (Table 5), it was possible to find at least one package leaflet of an equivalent medicine (i.e. the same composition, dosage and pharmaceutical form) available in the United Kingdom, United States and Australia. The exceptions were fosinopril 20 mg tablets and ramipril + hydrochlorothiazide 2.5 mg/12.5 mg or 5 mg/25 mg tablets (a mixed package leaflet), which apparently were not marketed in these three countries at the time of the study. These package leaflets were available for public consultation on the websites of the medicines agencies and health products authorities, respectively the Medicines and Healthcare Products Regulatory Agency (http://www.mhra.gov.uk/Safetyinformation/Medicinesinformation/SPCandPILs/), the Food and Drug Administration (http://www.fda.gov/Drugs/DrugSafety/ucm085729.htm) and the Therapeutic Goods Administration (http://www.tga.gov.au/consumers/information-medicines-cmi.htm#.UrMxNPRdV1Y). Similarly to the package leaflets for Portuguese medicines, omissions relating to dosage (both quantity and frequency of administration) were identified in the package leaflets of medicines from these three countries (Table 5).

In a subsample of the package leaflets (87 from non-steroidal anti-inflammatory drugs, 33 from angiotensin-converting-enzyme inhibitors and 27 from anxiolytics/sedatives/hypnotics), overdose symptoms were not described in respectively 36.8%, 24.2% and 11.1%.

## DISCUSSION

In the European Union, medicines authorities are responsible for approving and updating the contents of package leaflets (e.g. in the event of urgent safety measures being required).^3^^,^^26^^,^^27^ The scientific committees of the European Medicines Agency have developed and implemented diverse regulatory requirements in relation to medicinal products, such that manufacturers and marketing authorization holders have to observe these laws.^2^ The lack of scientific publications about package leaflets confirms that there is a need for further independent specific studies in this area. Moreover, evaluations of package leaflets performed outside the legal systems (e.g. in academic centers) seem to be scarce, despite the fact that they are clearly advisable for safety reasons.

In the present study, the checklist was considered to be a useful and workable screening tool for assessing compliance between the content of the package leaflets and some relevant regulatory issues. It covered critical issues regarding adequate use of the medicines^28^ and constituted an appropriate method for gathering data, taking into consideration the high number of package leaflets and the volume of information available. Although the Portuguese medicines were not all approved by a centralized procedure (under direct coordination from the European Medicines Agency), rather through mutual recognition or decentralized or national procedures (e.g. under the direct coordination of the National Medicines Agencies),^29^ almost all the package leaflets of the sample were developed in accordance with the six sections of the Quality Review of Documents for Human Product Information template,^2^ which is compulsory for package leaflets approved by a centralized procedure, for example.

### Mixed package leaflets

Naturally, patients consider that using package leaflets with more than one pharmaceutical form and/or strength is more difficult than using simple package leaflets (with only one pharmaceutical form and/or strength), because simple package leaflets present a lower degree of uncertainty regarding instructions on how to use the medicines.^30^ The possible high complexity associated with interpretation of mixed package leaflets means that it is advisable to increase the accuracy of the “end-user testing” (e.g. increasing the number of questions about dosage in face-to-face interviews).^30^^,^^31^ Even though the regulations of the European Medicines Agency recommend special care in approving mixed package leaflets,^30^ approximately one third of our sample were classified as mixed package leaflets. This situation might be explained by the fact that the production costs of the mixed package leaflets imply lower costs than those associated with the simple package leaflets*.* However, post-commercialization data specifically about safe use of mixed package leaflets are unavailable.

### Information on dosage (dose and frequency)

Omission of this essential information from package leaflets is considered controversial, because although package leaflets are one of the most accessible and reliable sources of drug information, some patients still prefer direct counseling from healthcare professionals.^13^ Nevertheless, it was possible to identify in this sample that there were some package leaflets without descriptions of the dose and frequency of administration, which are compulsory regulatory issues.^9^^,^^10^^,^^11^ Omission of dosage description might be considered acceptable in some cases, for example: 1) in the case of medicines for which administration is dependent on a healthcare professional’s intervention (e.g. injectable forms); or 2) in the case of complex and adjustable administration regimens, such as those commonly used for treating well-known chronic diseases, e.g. fosinopril 20 mg tablets and ramipril + hydrochlorothiazide 2.5 mg/12.5 mg or 5 mg/25 mg tablets for hypertension, because in these cases, the chronic patients (if trained) are capable of making the necessary adjustments to their own dosage, according to their blood pressure.

The qualitative analysis on the content of package leaflets of equivalent medicines commercialized in other countries also showed cases of both complete and incomplete information about the dosage. However, there were differences and some of these may be explained by the slight variances between the regulatory requirements of these countries (i.e. United States, United Kingdom and Australia). Hence, broader investigations on this issue are recommended.

### Information on adverse reactions

Since the publication of the first Quality Review of Documents for Human Product Information template in 1996, both descriptions of adverse reactions and dosage guidelines have been compulsory items in the content of package leaflets.^2^^,^^3^ More recently, as a consequence of publication of the Quality Review of Documents for Human Product Information template version 8,^2^ the required information on adverse reactions has become even more detailed. Serious adverse reactions now have to be listed first and these data need to be supplemented with clear instructions for patients on how to deal with them, thus confirming the medicines agencies’ interest in this issue.^3^

According to specialized publications, in the event of omission of data on the frequency of adverse reactions, patients might overestimate the risk and not adhere to treatment.^8^^,^^12^^,^^16^ Despite the cultural and literacy differences between the populations of different countries, the impact of information on the adverse reactions described in package leaflets has never been specifically evaluated in Portugal. Following the trends of other European countries,^2^^,^^20^ almost all of the package leaflets in the sample included a description of adverse reactions (a compulsory regulatory issue) and more or less half of the package leaflets in the sample described the frequency of adverse reactions (an optional regulatory issue).^2^^,^^10^^,^^11^ The omission of psychomotor limitations in some package leaflets of anxiolytic/sedative/hypnotic drugs, and also hypotension, dizziness and kidney and liver toxicity in some leaflets of angiotensin-converting-enzyme inhibitors might be considered to be an issue. These situations may possibly interfere with daily activities (e.g. driving) or pose other safety issues for patients with liver or kidney impairment.^24^

The lack of consistency between the contents of package leaflets of equivalent medicines can be considered to be troublesome, given the possible interchangeability of prescriptions between brands of medicine with the growth in market share of generic drugs.^22^ This might be explained by: 1) the existence of optional regulatory topics, thus allowing marketing authorization holders to decide whether to include certain types of information or not (i.e. optional regulatory issues); and 2) the fact that marketing authorization holders might deliberately not always provide complete information, for reasons such as the belief that the information is too intimidating,^1^ with the capability of influencing patients’ behavior, and consequently their health outcomes.

### Information on overdoses

The characteristics of overdose episodes vary from patient to patient, for instance with regard to: 1) the pharmacological properties of the drugs (e.g. narrow therapeutic margins may produce different negative events with slight differences in the doses); or 2) the pharmaceutical forms of the medicines (e.g. parenteral medicines because of their usual high and fast bioavailability).^24^

Updated content for package leaflets with regard to overdoses is considered necessary, since specific descriptions of signs and what to do in cases of overdose were not available in many of the package leaflets of the sample; however, the recommendation to consult a physician or pharmacist was present in the majority of the package leaflets. In contrast, it was possible to identify package leaflets of medicines with a low likelihood of intoxication (e.g. pyritinol) that specifically described the symptoms and procedures in cases of an overdose. Regarding the descriptions of overdose symptoms and the specific procedures in cases of overdose described in the package leaflets,^2^ the smallest number of omissions was found in the package leaflets of the medicines in Group 2 (central nervous system), probably because of their pharmacological properties and the high risk associated with use of medicines from this group (e.g. benzodiazepines).^24^ With regard to appropriate actions in cases of overdose, this information was also sometimes omitted from the content of the package leaflets in other countries (e.g. it was found in only 75.3% of 271 German package leaflets).^20^

### Information on the type of sentences

The updated nature and adequacy of the package leaflets in our sample was confirmed, given that all of them included at least sentence that was equal or similar (i.e. conveying an equivalent meaning) to those described in Table 2. This observation was based on the facts that all the sentences described in Table 2: 1) are defined in the Quality Review of Documents for Human Product Information template 7.3.1 (the template approved recently); and 2) are specific to the package leaflets of medicines approved through a centralized procedure.^2^^,^^3^

### Practical implications

In addition to the usability test recommended in the guidelines of the European Medicines Agency,^6^^,^^31^ both national and international medicines authorities should require the use of algorithms or other experimental methodologies (e.g. eye-tracking systems),^32^ to prove that the information included in the package leaflets is not only essential but also usable (e.g. package leaflets that are not too long).^20^^,^^21^^,^^33^ Contributions from all stakeholders are recommended with regard to developing new evidence-based guidelines (e.g. including the involvement of national patients), with the objective of assuring optimized intelligibility of the package leaflets within the specific cultural context of each country. On the other hand, standardization of more segments of the text should be considered, such as predefinition of the different parts of the text, depending on the nature of the active substances of the medicines. Given a higher degree of standardization, greater proportions of the package leaflet text should be organized for each active substance that has been officially approved by the medicines authorities, with publication in public databases. This information should be made mandatory as the minimum necessary in each package leaflet. Publication of standardized parts of the text in public databases constitutes an open regulatory procedure, which would be expected to actively contribute towards safe and effective use of medicines. Through application of the abovementioned measures, it will be possible to ensure that information in package leaflets of all therapeutic groups (e.g. from the central nervous, cardiovascular or musculoskeletal systems) is more accurate and complete, i.e. avoiding the differences within the obligatory or optional regulatory information that were detected in the present study. Overall, regulations regarding the readability of package leaflets should be updated with the objectives of standardizing the content of package leaflets and suppressing inconsistencies between the package leaflets of equivalent medicines.

## CONCLUSIONS

While the majority of the package leaflets of a representative sample of Portuguese medicines were developed in conformity with compulsory regulatory issues, a small proportion of these leaflets needed to be updated with regard to missing information within their content (e.g. descriptions of doses, schedules and adverse reactions) and variability in the information amongst equivalent package leaflets. The relevance of this updating is even greater considering that the missing information is often not covered during visits to physicians or pharmacists (e.g. descriptions of overdose symptoms or overdose management). Thus, the incomplete content of some of the package leaflets might increase the risk of unsafe use of the medicines by the patients. The problems identified in this sample of Portuguese package leaflets probably also exist in the package leaflets of other European countries, since some of them were direct translations from the original country producer leaflets.

Ideally, the methodologies for package leaflet approval should be optimized as a way of ensuring the inclusion of essential information for safe use of medicines, not only through application of guidelines but also by using experimental methodologies and algorithms, which in our case would involve specifically enrolling Portuguese patients. Furthermore, future research and market monitoring regarding the use of package leaflets are advisable, e.g. creating specific forms for health professionals or patients to communicate problems associated with use of package leaflets.
